# ‘A Story of Being Invisible’: A Single Case Study on the Significance of Being Recognised When Needing Acute Healthcare in the Early COVID-19 Pandemic

**DOI:** 10.1177/10497323231197375

**Published:** 2023-08-31

**Authors:** Nina Jøranson, Oddgeir Synnes, Anne Kari Tolo Heggestad, Grete Breievne, Marius Myrstad, Kristi Elisabeth Heiberg, Marte Meyer Walle-Hansen, Hilde Lausund

**Affiliations:** 1 Faculty of Health Studies, 87368 VID Specialized University, Oslo, Norway; 2 Centre of Diaconia and Professional Practice, 87368 VID Specialized University, Oslo, Norway; 3 Center for Medical Ethics, University of Oslo, Oslo, Norway; 4 Faculty of Health and Social Sciences, Institute of Nursing and Health Sciences, 11310 University of South-Eastern Norway, Drammen, Norway; 5 Department of Medical Research, 155273 Bærum Hospital Vestre Viken Hospital Trust, Drammen, Norway; 6 Department of Rehabilitation Science and Health Technology, 158935 OsloMet - Oslo Metropolitan University, Oslo, Norway

**Keywords:** older people, experiences, COVID-19, healthcare personnel, storytelling, dialogical narrative analysis, health promotion

## Abstract

Severe illness is often an existential threat that triggers emotions like fear, stress, and anxiousness. Such emotions can affect ill patients’ encounters with healthcare personnel. We present a single case study of an older woman who contracted COVID-19 and her challenge to be recognised by healthcare personnel in the early pandemic. Storytelling is vital to understand how patients can create meaning in illness as it gives them the opportunity to reshape and restore their past and to project a future. We used Arthur Frank’s *dialogical narrative analysis* to explore how one patient experienced her encounters with healthcare personnel. Although she felt very ill from COVID-19, she experienced being almost invisible and not being believed by healthcare personnel in a system marked by high stress levels and uncertainty. Despite rejections and illness, she managed to mobilise her resources, even though she depended on significant others. Her story brings forward altered self-understanding and growth. The importance of facilitating dialogical settings for healthcare professionals through patient storytelling also contributes to a broader societal understanding of illness beyond a biological perspective.

## Introduction

During the first phase of the COVID-19 pandemic, a national lockdown was implemented in Norway on 12 March 2020. The primary aim was to prevent an unmanageable number of critically ill patients in need of intensive care. The need to avoid overburdening the healthcare system was communicated broadly by the national authorities (NOU2021:6), but hospitals in the Oslo region experienced a steep increase in admissions with COVID-19 during the first three to four weeks of the lockdown (Ihle-Hansen et al., 2020). The extraordinary measures that were introduced entailed complex challenges for healthcare professionals in various settings (NOU2021:6). Infected patients had contracted an unknown and experienced a frightening disease with unfamiliar and severe symptoms. Uncertainty regarding outcomes of COVID-19 was a troubling backdrop to their search for medical help (Jøranson et al., 2022).

Patients’ experiences underscore that severe illness is an existential threat to human beings. People with severe illness often develop a feeling of being alienated from their body and losing control, to fear a twist of hopes and dreams, and their existence seems to be out of balance. Illness alters how we experience our world. These perceptions also affect the perspectives of the doctor and the patient during examination for an illness (Svenaeus, 2011). When patients are suffering from illness, their display of fear, stress, and anxiety can affect their relationship with healthcare personnel, and mutual respect may not be present due to lack of the basic conditions necessary to create successful communication and relationship in the setting (Blakar, 2013). If providers ignore the patient’s story, it may lead to important aspects of the patient being overlooked if only physiological symptoms are to be examined to determine a diagnosis (Svenaeus, 2011).

To bring forward further insight into how severe illness may affect vulnerable patients and their encounters with healthcare personnel, the use of storytelling can help us understand patients’ experiences. According to the medical sociologist Arthur Frank (2013), stories are vital for patients to create meaning when they face severe illness. Illness represents a biographical disruption, and stories might help sick people create a new understanding of their situation and thus convert from understanding their illness as a destiny to understanding it as an experience by reshaping their past and imaginatively projecting a future (Frank, 2012, 2013). Hence, stories might allow people to revise their sense of self (Frank, 2010, 2012). At the same time, stories are never told in isolation and are always influenced by the larger stories of society and the available narrative resources in a culture. The narratives ill persons tell are thus also a means to understand illness beyond a personal or medical perspective and can elicit understanding of illness in a sociological and cultural context (Frank, 2013; Hydén, 1997). Several studies described storytelling in illness as a means of coping with a condition, for instance, living with chronic illness (Cangelosi & Sorrell, 2008; Hydén, 1997; Synnes et al., 2020).

The aim of this article is to explore how a single patient suffering from COVID-19 experienced and narrated encounters with healthcare personnel during the first phase of the pandemic.

## Methods

### Study Design, Setting, and Participant

This is a single case study which was part of a larger multicentre cohort study that aimed to investigate change in quality of life, functional decline, and mortality of older people six months following hospital admission due to COVID-19 (Walle-Hansen et al., 2021). The main cohort study, GeroCovid, was planned by geriatricians in several EU countries and Norway at the beginning of the pandemic, with an observational study design without experimental interventions (Trevisan et al., 2021). The study was supported by the Norwegian Geriatric Society.

We conducted a qualitative sub-study to explore and describe older patients’ experiences with having COVID-19 before, during, and after hospital admission, from which this single case is taken. The sub-study has been described previously (Heiberg et al., 2022a, 2022b; Jøranson et al., 2022).

The informant, a woman we call Anne, is in her 60s and is a certified healthcare professional, married, and with close family relations. During the interview, we discovered her need to tell what was still an inchoate story which needed to be given a narrative form through her storytelling. We found this particular informant’s descriptions to be striking, containing rich elaborations of her dramatic experiences with being infected with and developing symptoms of COVID-19, and with an experience of not being recognised by healthcare personnel during her search for healthcare. By her own choice, she was interviewed in a meeting room at the university, and the session lasted for almost 2 hours. The interview was audio recorded and transcribed by an external secretary.

### Data Construction

During the interview, conducted by the first and third authors, we were struck by Anne’s animated story and her willingness to tell it. She captivated us with her story, and early in the interview, we chose to lay aside the interview guide to grasp what was at stake for the storyteller. We wanted to explore the story she told us rather than relate to the semi-structured interview guide. The storyteller had a lot on her mind, and our dialogical conversation lasted almost twice as long as the other interviews. According to Frank (2012), dialogical narrative analysis involves selecting stories on the basis of *phronesis*, meaning that stories are chosen based on an intuitive sense – or practical wisdom; our intuitive sense was built on our previous experience as researchers. When a story touched us, like Anne’s story did, we knew we had to listen attentively. We did not choose her story to represent reality as it is but rather to bring forward her reality ‘as [she] need[ed] to tell it’ (Frank, 2012, p. 38). During the interview, she also used her diary notes to check chronology, and she showed us cell phone pictures of her visual symptoms to emphasise details from her story. When reading the interview, we realised her ability to bring forward rich, complex, and comprehensive descriptions from her experiences. After witnessing Anne’s storytelling, we almost felt obliged to bring her story forward.

### Narrative Analysis

To grasp and investigate the informant’s meaning making and her illness experience in our interpretation of the dialogue, we chose to perform a dialogical narrative analysis as described by Frank (2010, 2012). In dialogical listening, there are usually three elements in play: the story, the storyteller, and the listener. The latter listens attentively to grasp the whole story being told, to practice a sustained tension between dialogue and analysis (Frank, 2012). Further, Frank emphasises that we must let the story breathe – meaning that the exploration of the story should be ‘less about finding themes and more about asking what stories do, which is to inform human life’ (Frank, 2010, p. 2). The dialogical narrative analysis started in the interview situation and continued during the reading and re-readings of the transcribed interview. In narrative analysis, the whole account is looked at as a meaningful unit in the analysis, instead of being ordered around themes and categories, for the analysis to remain truly narrative (Riessman, 2008). In Frank’s dialogical narrative analysis, it is proposed to ask overarching questions about the stories that can help us understand what is at stake for the storytellers in their narration. In our analysis, we are thus concerned with the following question from Frank’s analysis: How is the storyteller ‘holding her own’ in the act of storytelling? By ‘holding one’s own’ Frank means ‘seeking to sustain the value of oneself or identity in response to whatever threatens to diminish that self or identity’ (Frank, 2012, p. 33).

In our analysis, we have thus let the following question guide our reading, based on what we experienced was central in the storytelling moment, as well as what we found crucial in the analysis of the transcript:

What is at stake in Anne’s story in her struggles for healthcare?

In reading Anne’s story, we find that what is at stake is her struggles of being believed: firstly, in her search for healthcare and secondly by telling a story that can capture her struggles and experiences. When reading the transcript, we saw that Anne’s fight to be believed about having contracted COVID-19 is closely connected to her distinct fear of dying and her gratitude to live. By looking at narrative units in the story, we saw how Anne strived to create meaning and make sense of her situation based on the above-mentioned aspects of her story. To add further insight into the overarching research question, we also asked some subsidiary questions that underscore a holistic reading of the narrative material (Frank, 2012, 2013; Riessman, 2008):

• What are the significant events in the story?
• Who are the central characters in the story?
• What different voices of self are found in the story?

## Anne’s Story

In presenting the findings, we highlight the main issue for the storyteller: ‘her struggle to make herself believed’. Through her recounting of several encounters, we hear how the narrator tried to find ways to make the people believe that she had contracted COVID-19 and that her severe symptoms required treatment. In this pursuit, she called upon her own resources, her family, and her surroundings.

We present findings two-fold to answer the overall research question. Firstly, we present the condensed story in which Anne conveys her experiences seeking healthcare in various situations. Secondly, we present the findings to be analysed in light of the three narrative analytical questions, which underscore crucial aspects of the meaning making of our storyteller.

Anne was infected with the virus SARS-CoV-2 during the very early phase of the pandemic in Norway. Within a few days, she developed several symptoms, including a high fever, a pounding headache, coughing, dyspnoea, and muscle rigidity. As an experienced healthcare worker, she recognised that the symptoms were different from the flu, and she felt that something was seriously wrong. Anne believed she had COVID-19: ‘I don’t know how to explain this, but the symptoms were massive in my whole body. It was so, the beginning was so rough. I didn’t have one particular symptom’.

She called the official COVID-19 helpline, and after waiting for several hours on the phone, she described her situation but was dismissed by the responding nurse:

The nurse was absolutely certain that since I was under 80 years of age, had not visited Austria or travelled to other countries, really nothing, no underlying disease, that I could not possibly have COVID-19. That was just how it was.

Anne didn’t know how she got infected because she had not been abroad. However, in hindsight, she realised that the world had come closer to her via an accidental close contact with a tourist group during a domestic flight.

Anne described in detail how she experienced the course of the disease. Due to the rapid and serious development of her illness, she visited several places offering medical help, including emergency rooms and her general practitioner’s office. Although she explained her symptoms and pleaded for a SARS-CoV-2 test, her statements were rejected by the doctors. They discovered other infections to treat, with one doctor diagnosing severe sinusitis, although he could not help her beyond prescribing antibiotics: ‘I can’t refer you to the hospital, but there is something wrong with you’.

All the doctors she encountered said that she could not possibly have COVID-19 if she hadn’t been abroad because there was no SARS-CoV-2 infection in Norway yet. Anne felt despair at not being believed and developed a growing fear of dying as she felt her symptoms worsening. As she developed more symptoms, she also decreased her nutritional intake. After a couple of weeks, she was throwing up almost everything she ingested, including medication, which she felt worsened her general condition and drained her of energy. She continued her quest for healthcare. During a new emergency room examination, she was observed by a second doctor, who, she recounted, recognised her displayed symptoms as COVID-19. Anne told how this doctor, by chance, had a test in the pocket and offered it to her, albeit discreetly. She described this doctor as reasoning differently than the other doctors: ‘This was a human being who, for some reason, recognised me. I don’t know about the others’. Anne reflected upon how this doctor ignored the national testing criteria and chose to focus on the patient’s experiences and symptom descriptions, an approach which fascinated her: ‘The doctor reasoned differently and broke [the guidelines] and risked something. Isn’t that interesting?’

The positive test released medical help and in-home visits from a doctor. It was now almost four weeks since she had contracted SARS-CoV-2, and Anne felt severely ill and exhausted. She struggled to breathe and still had a high fever and a pounding headache, symptoms which horrified her of how the disease was ravaging her body. Anne cried while telling us about this visiting doctor, who she experienced actually recognised her by expressing great concern about her lung function and dismay that she was alone in such a severe state. An ambulance brought her to the COVID-19 ward at the hospital.

Shortly after being admitted to the hospital, Anne finally managed to eat a slice of bread without vomiting. In addition, her oxygen saturation was slowly improving, so the doctors considered her condition as well enough to be sent home the next day. Anne felt annoyed that these doctors did not take her symptom experiences and the short treatment period into account. She tried to protest but experienced only a brusque dismissal. The next day she tried to negotiate with the discharging doctor by mobilising some of her recovered strength. Again, she didn’t get through: ‘There was no more to discuss on the matter. The doctor just walked away. Oh dear! Just walked away’. Being sent home terrified Anne because she felt exhausted and unable to take care of herself. Drained of energy, she resigned herself to the hospital bed.

After discharge, Anne wrote in her diary that very night: ‘Sent home to die’. She thought she wouldn’t survive the night. She was alone in her house, still wearing her outdoor coat; her symptoms had exacerbated, and she experienced confusion and periods of unconsciousness. Outside the house was her family, who suspected she was seriously ill because she did not respond to their numerous calls. They ordered an ambulance, which brought her with sirens back to the same hospital ward.

Anne woke up in the ward and realised that she would live on. She told us that she recovered relatively fast, although she still felt weak. She was discharged after 5 days.

When we met Anne half a year later, she believed she most likely had developed late complications from COVID-19 (long COVID) in the form of cardiac issues, reduced respiratory capacity, fatigue, and some short-term memory challenges. When looking back at her dramatic experience, she reflected on how unprepared the healthcare system seemed to face this unknown disease and pandemic conditions. These reflections were taken into account when she reviewed some of the highly uncomfortable encounters she experienced: ‘(...), I believe that healthcare staff back then must have received quite strict instructions regarding not burdening the system. I believe they must have been scared, otherwise, you couldn’t be as vicious as some of them seemed’.

The most striking element in her story is the metaphor Anne chose to use to sum up her experiences and feelings related to all the dismissals she encountered when she felt like a severely ill patient. She called it ‘(…) an absurd Kafka-experience’:

It is just like standing inside a metal box that is transparent and yelling ‘I’m dying!’ And everyone is standing around on the outside, eating and drinking. They don’t notice that there is anyone in there. A completely insane experience!

During the course of COVID-19, her dramatic experiences made her appreciate life to a greater extent and gave her new and significant life perspectives in the aftermath. She was so pleased to have developed stronger feelings of inner safety and calmness, and she told us she would no longer waste energy on insignificant worries. She reflected more consciously on the meaning of life and the goodness in it: ‘Death is there. We know it, but we shall not [die], the earth is so beautiful, it is so beautiful here. I think of this every day. This is where we are. This is what we should love’.

Towards the end of the interview, she summed up her dramatic experience of feeling almost invisible as a patient in terms of not being taken seriously or believed about her symptoms, despite her resources. Following her reflections on the course of the disease and her encounters with healthcare staff, she was still puzzled as to how difficult it was to reach through and be seen in these encounters. As she summed it up, ‘this is a story of being invisible when we are so close to each other’.

### Analytical Interpretation of Anne’s Story

To assess analytically what kind of illness story Anne told us, we started by looking at Frank’s typology of illness stories. According to Frank (2013), most illness stories display aspects of *chaos*, *restitution*, and *quest*. Anne’s story contains traces of these three elements. *Chaos* appears in the lack of acknowledgement from healthcare personnel and in the absurdity surrounding Anne’s struggles to find understanding and a treatment for her condition, what she terms a ‘Kafka trial’. Hers is also a story of *restitution*, as Anne does indeed get well after finally having received treatment. But we regard her story primarily as a *quest* story: Anne recovered from her illness, and despite feeling marked by long COVID, her experience seemed to make her feel reorientated and reborn, almost like a Phoenix who had risen from the ashes. Looking back, she had altered her self-understanding, and she wanted to share her new insights with others through her story. This demonstrates what Frank terms a *quest narrative* (Frank, 2012, 2013), where the storyteller constructively develops experiences into personal learning and growth, an insight that must be passed on to others.

When we take into account the insight Anne conveys in her narrative, the plot revolves around her experiences of not being believed to have contracted a serious illness or have serious bodily symptoms, which together describe how she experienced not being seen in these encounters. To Anne, the fear of dying from this unknown and most likely severe disease was also at stake.

Ahead of her positive test, only one doctor was willing to test her, and even then, only discreetly. This action was of great significance to her and is a turning point in the story. Soon after, there was a new turning point when she was discharged, although she felt unable to take care of herself. That very night she believed she would have died if it wasn’t for her family’s determined actions, which to Anne were highly significant and represented another turning point. An additional significant event was when Anne woke up in the ward and realised that she actually would live on.

A story consists of significant characters. In Anne’s story, two of the central characters were doctors, namely the one who tested her and the one who examined her at home before her first hospital admission. She was deeply moved when she talked about them, and their actions were highly significant to her. In addition, her family was extremely important throughout the course of her illness, and their actions were probably decisive for the outcome. The *quest narrative* is oriented towards others to learn from the storyteller’s reorientations. A central tension in Anne’s story is between the central characters, namely the doctors and her family, who are the central helpers in her story, and the doctors who decided to discharge her from the hospital, who play the role of antagonists.

We can also hear several voices of self in Anne’s story when she called upon her own resources during the course of the disease. Anne presents herself as strong-willed and able to negotiate and stand up for herself and to imagine and find solutions to future obstacles. She quickly understood that she must have contracted COVID-19. As a certified health professional, she knew the significance of a positive test towards the strict gatekeepers in the emergency rooms. She struggled to argue with the hospital doctors to let her stay in the ward, despite her weakened state. She called upon her family, who stood up for her. The extent of her own inner resources, in addition to her family’s support, seems to be important to the outcome of her story. Anne also called upon her new insights, which helped her in dealing with long COVID. She also reflected to try to understand why healthcare personnel had to be strict gatekeepers. When listening to Anne’s rich story, we were intrigued to learn how a human being who had had such a dramatic experience could display so much hope and gratitude and such a zest for life in the aftermath. Her comments on the beauty of life illustrate a central trait of quest narratives: how illness might involve a newfound capacity for growth and wonder. Anne’s quest narrative underpins her capacity to find ways of being strong and surviving. However, the story also contains her vulnerable sides, including her terror due to development of serious symptoms and her increasing desperation because of not being believed to be in need of healthcare. As a storyteller, Anne also displayed the vulnerable feelings attached to some of her most painful experiences, for example, when she wept while talking about caring doctors who believed her and showed anger while telling how she feared for her life after the first discharge.

The final reflection relates to Anne’s description of her experienced dismissals as *‘*‘*a Kafka trial*’, a phrase she used twice during the interview to sum up her experiences of not being seen during the encounters.

This interview was the first time she had told her whole story to anyone, and she found it relieving. She believed the storytelling did her good, so much so that she almost didn’t want to leave us for her next appointment. She expressed both gratefulness and usefulness to finally be able to construct ‘*a kind of a structure of the story*’*.*

## Discussion of Anne’s Story

Anne’s story is an example of how stressful unknown illness can be to a person who experiences several rejections as a patient in need of healthcare. We asked the overall research question: *What is at stake in Anne’s story in her struggles for healthcare?* When analysing her story, we identified being recognised as the main theme. Anne’s story shows the significance of being a vulnerable patient, and her efforts during several encounters with medical personnel, the support she received from significant others, and her communication with healthcare personnel in different settings all illustrate various ways of being recognised. In addition, the story shows how she reflects on her quest for healthcare. It is by hearing how Anne interprets the main events in her story in light of her previous life stories and how she draws on available resources in and around herself that we are able to grasp how she found the means to come out stronger after her illness with COVID-19.

### Stressful Relations in Complex Contexts

Anne told us about the strange symptoms that invaded her body, and when she realised that she must have contracted coronavirus, she became scared and needed clarification, such as a test. Having an illness with symptoms will often induce a suffering human to seek medical help (Svenaeus, 2011). During an ideal medical assessment, the knowledgeable, responsible, and reflective medical doctor, who is an expert, will examine the patient, who is an expert about their own body, problem, and life situation. For each expert to share their expertise in this setting to create an expedient relationship, it requires mutual respect based on an ability to set aside one’s own perspectives; this is expected of the medical doctor in particular (Blakar, 2013; Svenaeus, 2011). This perspective corresponds to the performance of a dialogical conversation. Dialogue presupposes mutual recognition between the involved parties, as well as a willingness to share (Frank, 2002, 2005). However, scenario of a knowledgeable medical doctor assisting a vulnerable patient will necessarily entail a profoundly asymmetrical relationship (Deal, 2011; Schei, 2013); Anne experienced this dynamic as monological due to the medical expressions allowed from the doctor. Considering the context of the early pandemic, stress levels were high due to unpredictability and uncertainty (NOU2021:6), which most likely led to a lack of the basic conditions necessary to create the kinds of successful relations that are essential to establishing dialogue. In Anne’s case, the unbalanced provider–patient relationship was probably reinforced by the stress level, and the conversation became a monologue.

Regardless of a context affected by the pandemic and stressful examinations, reflections on practice and the relation between patient and healthcare personnel are crucial. Due to their status and their role as gatekeepers for healthcare services, doctors possess authoritative power, which affects people’s thoughts, perceptions, and behaviour; this power is also recognised by society. However, the doctor’s personal values and norms, usually portrayed as knowledge, are also a form of power and as such affect relations with patients. This dynamic underlines the importance of competence in patient communication, including having an informed mind, to prevent disrespect in relations (Schei, 2013; Svenaeus, 2011), particularly when the case doesn’t fit the prevailing medical narrative.

The relational interaction in dialogical conversations is influenced by contextual aspects. In Anne’s case, her doctors’ personal values, norms, and knowledge played a role not only in the interaction itself but also in Anne’s story as a whole. Context is not just organisational structure or physical entities but includes features that shape relations and interactions (May et al., 2016), and identifying contextual aspects of relational interactions contributes to describing the complexity of practice (Nicolini, 2012). Anne made several attempts to access necessary medical information and/or treatment but struggled to pass the strict gatekeepers as she didn’t meet ‘the strict national corona criteria’ (Jøranson et al., 2022). The challenge of testing ill patients and the adherence to strict priorities due to fear of overloading the hospital wards are other contextual aspects in this story, and these were communicated strictly monologically from the macro level to be handled on a micro level by healthcare personnel (NOU2021:6). However, prioritisation was difficult due to limited knowledge of COVID-19, an escalating number of reports about the spread of a scary infection, and assessments, all contextual factors affecting the triage of patients. Expectations from the macro level propagated downwards, most likely making already asymmetrical relationships even more skewed during providers’ encounters with patients in need of medical help. Such unpredictable factors made the context even more complex and may have influenced Anne’s experience of not being recognised as a patient with severe symptoms on the micro level.

Anne’s story revolves around dismissals by health professionals in various medical encounters where she experienced both being believed to be ill, although not with COVID-19 and, later, not being recognised as severely ill. These dismissals underscored her fears, in what she called a *Kafka trial*: She experienced that no one recognised her condition. *Kafka trial* is derived from Kafka’s ‘The Trial’ and is a metaphor characterised by something as being bureaucratic, threatening, and unreal (Dictionary of the Norwegian Academy, n.d.), and Anne’s story contains these elements. The bureaucratic, threatening, and unreal features are also contextual aspects influencing Anne’s experience. During the storytelling, we see how Anne called upon her reflective resources in the aftermath of her illness by using the Kafka trial as metaphor to frame her most horrible feelings from her experience with COVID-19.

Another feature of contextual value in the story is that healthcare personnel also were inflicted with a vulnerability, by virtue of the unknown, which was supposed to be kept in check through low hospital admission rates (NOU2021:6). Consequently, relations and communication during encounters between Anne and healthcare personnel became very challenging. Encounters between patients and providers early in the pandemic were characterised by lack of knowledge on how to treat COVID-19, nervous patients, stressed healthcare personnel, lack of overview, etc., factors which probably affected most settings and relations. In addition, healthcare personnel struggled to conduct triage in accordance with prioritisation criteria to ensure fair and just allocation of healthcare (Jøranson et al., 2022; NOU2021:6).

### The Significance of Telling Your Story

Telling your story, including to healthcare personnel, can contribute to shape meaning when encountering severe illness and alter perceptions in a dialogical setting (Frank, 2013; Svenaeus, 2011). In addition, healthcare personnel can learn by listening to and reflecting on patients’ stories about their experiences with critical illness, illness perspectives, and what aspects of caring they value (Gurney et al., 2020). The sick person who turns their illness into a story can alter their perspective, from seeing illness as a destiny to seeing it as an experience, as in Anne’s story. When a story is told through a wounded body, storytelling will bring a new perspective for comprehending the illness (Frank, 2013). This contrasts with the usual illness perspective, in which stories are often told through medical lenses by doctors. The lens through which a story is told, however, enables the storyteller to create meaning from illness, rather than to be a victim of disease. This might also contribute to a feeling of coherence in mastering the illness. Giving storytellers an arena enables them to influence healthcare personnel’s comprehensions of illness, which Frank (2013) calls ‘the pedagogy of suffering’. Stories can pave the way for more symmetrical care relations – and the recognition that we all need to be part of communities. They can also show the complexity of situations and how it shapes interactions. In addition, ‘the pedagogy of suffering’ is a way to encourage a broader comprehension of illness in society that goes beyond the traditional physiological illness perspective. ‘By conceiving suffering as a pedagogy, agency is restored to ill people; testimony is given equal place alongside professional expertise’ (Frank, 2013, p. 145).

Mastering the stressors of illness and giving illness meaning requires the ability to comprehend one’s illness situation and to find the capacity to harness available resources. This capability is central in coping strategies, especially from a salutogenic perspective: People use their capacities to assess and comprehend their situation, to find meaning, to move in a health promotive direction, and to manage their health, all to develop a sense of coherence to master stressors and create wellness (Antonovsky, 1987). Such strategies are widely used to cope with illness and are decisive for creating wellness from illness (Haugan & Eriksson, 2021). By telling her story, Anne called on her resources and seemed to find ways to comprehend, understand, and manage her situation to alter her illness into experiences. She found ways to be recognised, despite the dismissals she had experienced, when she mobilised her new insights and resources post-COVID. She developed new capacities to manage long COVID in addition to expressing joy and gratitude for what she saw as the meaning of life. Anne’s actions are in line with the perceptual mechanism involved in developing a sense of coherence, which suggests that to deal with stressors, people need to reflect on their situation using available resources to feel empowered (Super et al., 2015).

Anne experienced not being allowed to tell her story to healthcare personnel during the course of her illness. She was, however, given the opportunity to tell *us* her whole story. She underlined how relieved she was to finally put all the bits and pieces together, which she did by constructing a precise sequence of events to frame her experiences and feelings. The moral purpose of reading quest stories is ‘to witness a change of character through suffering’ (Frank, 2013, p. 128). Anne shared her experiences and reflections, new insights, growth, and learning, which exemplify the significance of being given an opportunity to revise her sense of self through storytelling (Frank, 2010, 2012). According to Frank (2013), stories need an audience, which implies a relation between the storyteller and the listener, to reach their inherent potential to affect others. This highlights the need for medical doctors and other healthcare professionals to develop a ‘narrative competence’ (Charon et al., 2016) that can acknowledge what is at stake in patients’ narrations and the important role healthcare professionals play in facilitating or hindering stories. Supporting patient-centredness in healthcare thus ought to open towards collaborative dialogues of narratives between patients and professionals (Josephsson et al., 2022).

## Strengths and Limitations

It is not our aim, nor is it possible, to generalise from Anne’s story. Our objective was to highlight different aspects of her story to learn more about the complex personal experiences that can emerge when contracting an unknown and dangerous disease and in the quest for healthcare, and to facilitate dialogical approaches. Anne is a unique case, but her experiences can have a wider relevance for other patients in need of help. The results from the study must therefore be regarded in terms of transferability rather than generalisability. Parts of Anne’s story will be recognisable to others, and many of the aspects of this story discussed in this article could also pertain to other situations besides the COVID-19 pandemic.

It is important to bear in mind that we only have access to Anne’s story, which stands unchallenged. When trying to understand both her perspective and how healthcare personnel acted during the encounters she describes, we should also consider that the story is told in retrospect. The findings must therefore be interpreted with caution.

## Conclusions and Clinical Implications

The main purpose of this study was to explore the story told by one single patient who experienced COVID-19 during the very first phase of the pandemic. Insights from stories may be of importance to healthcare professionals for understanding patients’ experiences and their perspectives during encounters by privileging dialogical settings. Establishing relations between providers and patients can be challenging, and it became all the more challenging early in the COVID-19 pandemic due to the stressful and unpredictable context created by an unfamiliar disease. However, privileging dialogical examinations and conversations by letting patients tell their stories will go a long way towards recognising their perspectives during encounters in various settings. Telling stories in dialogical encounters might function as a health protective factor, creating coherence and increasing wellbeing for the storytelling patient. In this study, the storyteller painted a whole picture for us, a picture that healthcare personnel were not able to grasp in a chaotic and unpredictable context.
